# Differentiating the relationships between traditional and new media use and sleep quality during the COVID-19 pandemic: roles of psychological distress and age

**DOI:** 10.3389/fpsyg.2024.1422004

**Published:** 2024-06-26

**Authors:** Tong Xu, Dongmei Zhu, Zhaoliang Yu, Junhua Dang, Helgi Schiöth

**Affiliations:** ^1^School of Education, Jianghan University, Wuhan, China; ^2^Department of Psychology, School of Humanities and Social Sciences, Fuzhou University, Fuzhou, China; ^3^Institute of Social Psychology, School of Humanities and Social Sciences, Xi’an Jiaotong University, Xi'an, China; ^4^Department of Surgical Sciences, Uppsala University, Uppsala, Sweden

**Keywords:** sleep quality, media use, COVID-19, psychological distress, age

## Abstract

**Background:**

Numerous studies have consistently demonstrated a decline in sleep quality during the COVID-19 pandemic. The primary objective of this study is to explore the impact of engaging with pertinent epidemic information through the media amid the COVID-19 crisis on individuals’ sleep quality and the underlying mechanisms through which this influence operates.

**Methods:**

An online cross-sectional study design was employed. A total of 1,063 British adults (36.2% males; *M*_age_ = 38.85, *SD*_age_ = 13.36, ranging from 18 to 77 years old) participated in the study and completed our questionnaires, which included media usage frequency during the pandemic, the 10-item Kessler Psychological Distress Scale (K10), the Insomnia Severity Index (ISI), and the Ten-item Personality Inventory (TIPI).

**Results:**

Pearson’s correlation analyses indicated that there was no significant correlation between COVID-19-related traditional media use (television, radio, newspaper) and psychological distress or sleep quality. However, exposure to information related to COVID-19 through new media use (Facebook, Tik Tok, Twitter) was correlated with greater psychological distress and poorer sleep quality. A moderated mediation analysis showed that psychological distress fully mediated the relationship between new media use and poor sleep, which was moderated by age, with the association between psychological distress and poor sleep quality being stronger among older adults.

**Conclusion:**

Exposure to information of COVID-19 via new (but not traditional) media use deteriorated sleep quality through greater psychological distress, and this relationship was stronger among older adults.

## 1 Introduction

In early 2020, the rapid global spread of COVID-19 led to widespread home isolation, resulting in varying degrees of sleep disturbances (Jahrami et al., 2021). Research indicates a significant increase in Google searches related to sleep issues during the COVID-19 pandemic compared to the period before, with a peak in queries around 3 a.m. in the United States (Zitting et al., 2021). The impact of sleep problems following the COVID-19 outbreak across different countries should not be underestimated, as the pandemic has exacerbated insomnia rates and compromised sleep quality (Jahrami et al., 2021). For instance, surveys conducted in several provinces and cities in Canada revealed a surge in insomnia rates from 15.5% before the pandemic to 17.7% during the outbreak, with 32.6% of individuals developing insomnia post-outbreak (Morin et al., 2022). Past literature revealed that several factors could contribute to disrupted sleep patterns during the pandemic, including intensified anxiety (Kang et al., 2020), reduced outdoor activities (Mochón-Benguigui et al., 2021), and increased screen time (Zhou et al., 2020).

Meanwhile, during times of epidemic outbreaks, individuals faced significant uncertainties due to highly infectious diseases, prompting them to engage in information-seeking behaviors to reduce uncertainty, form a coherent understanding of the disease, and guide their actions (Li et al., 2022). Home isolation and remote work, however, resulted in limited ways of accessing information, making media use one of the most frequently used channels to acquire information related to the disease’s severity, vaccine progress, and related updates (Conroy et al., 2021; Kanchan and Gaidhane, 2023). Therefore, it is pertinent to examine to what extent and how COVID-19 related media exposure might influence sleep quality during the pandemic.

### 1.1 COVID-19 related media use and sleep quality

Individuals are generally more susceptible to negative information (Baumeister et al., 2001), leading to anxiety and stress, subsequently affecting their sleep quality (Gao et al., 2020). Prolonged exposure to negative information can harm mental health, affecting sleep patterns (Choi et al., 2017). It has been found that prolonged exposure to negative media content could elevate anxiety and depressive symptoms, which in turn negatively predicted the depth and quality of sleep (Taylor and Blackford, 2020). Media consumption can also disrupt sleep quality by interfering with individuals’ circadian rhythm. With many people working or studying from home during epidemics and limited recreational activities, reliance on media for entertainment increases. However, prolonged use of electronic devices can disrupt sleep patterns, particularly with blue light radiation inhibiting melatonin secretion, impacting the ability to fall asleep and overall sleep quality (Chang et al., 2015). Excessive media exposure during epidemics can affect sleep quality for various reasons. For instance, overuse of social media may immerse individuals in a virtual world, diverting attention from real-life issues and affecting sleep (Scott et al., 2019). Furthermore, using various media platforms may lead to sleep disorders like insomnia, further impacting sleep quality (Cellini et al., 2020). Therefore, media consumption significantly influences sleep quality during epidemic periods, highlighting the need for further research and awareness of the impact of media exposure on individuals’ sleep patterns.

### 1.2 Psychological distress as a mediator

Psychological distress encompasses a range of emotional disorders such as depression, stress, and anxiety, reflecting an individual’s mental health status. Research indicates that psychological distress can escalate from common feelings of sadness, vulnerability, and fear to more severe mental health issues like depression and anxiety, significantly impacting an individual’s psychological well-being. During the COVID-19 pandemic, a notable positive correlation exists between psychological distress and poor sleep quality. Negative emotions play a pivotal role in the deterioration of sleep quality and the onset of sleep disorders. Consequently, psychological distress may indeed influence individuals’ sleep quality during the COVID-19 period.

In times of disasters, the media serves not only to disseminate essential disease-related information but also generates adverse effects. Prolonged exposure to media coverage of traumatic events can induce psychological stress reactions in audiences akin to those directly affected by the trauma. The continuous amplification of negative information’s psychological impact and the sharing of emotional responses on social media platforms can breed resistance and even irritation, leading to heightened psychological distress. Therefore, this study posits that psychological distress acts as a mediating factor between media exposure and sleep disturbance, emphasizing the intricate relationship between media consumption, psychological well-being, and sleep patterns.

### 1.3 Age as a moderator

While media exposure can impact sleep quality through psychological distress, certain individuals may be more susceptible to such effects. For example, research indicates that older individuals are more prone to experiencing sleep disturbances during the COVID-19 pandemic (Gao et al., 2021). The quality of sleep tends to decline with age, and older adults are particularly sensitive to negative influences such as illness, medications, and psychological stress (Li et al., 2018). This is further supported by Trabelsi et al. (2021), who observed worsened sleep quality among older adults during the COVID-19 pandemic, while Wang J. et al. (2020) demonstrated that older adults were more likely to report sleep disturbances compared to their younger counterparts during lockdowns in China. Unlike emotional well-being, older adults’ sleep may be more significantly affected by stress stemming from the COVID-19 pandemic than that of younger adults. A study conducted in October 2020 also revealed that older adults’ anxiety and depressive symptoms were more strongly associated with COVID-19-related stress than those of younger adults (Pearman et al., 2021), indicating heightened vulnerability in older adults due to prolonged pandemic stress. Therefore, the impact of psychological distress on sleep quality is likely to be moderated by age, suggesting that the influence of media exposure on sleep quality through psychological distress may be more pronounced in older individuals compared to younger individuals.

### 1.4 Research questions and hypotheses

The current study aimed to examine the mediating role of psychological distress and the moderating role of age between exposure to information of COVID-19 and sleep disturbance in UK. Based on the above literature review, this study proposed three hypotheses.

*Hypothesis 1*: Media exposure to information of COVID-19 should be positively related to sleep disturbance.

*Hypothesis 2*: Psychological distress mediates the relationship between exposure to information of COVID-19 and sleep disturbance. Specifically, media exposure to information of COVID-19 should predict increased psychological distress, which in turn predict higher levels of sleep disturbance.

*Hypothesis 3*: Age moderates the relationship between psychological distress and sleep disturbance, such that this relationship is stronger for older people.

**Figure 1:**
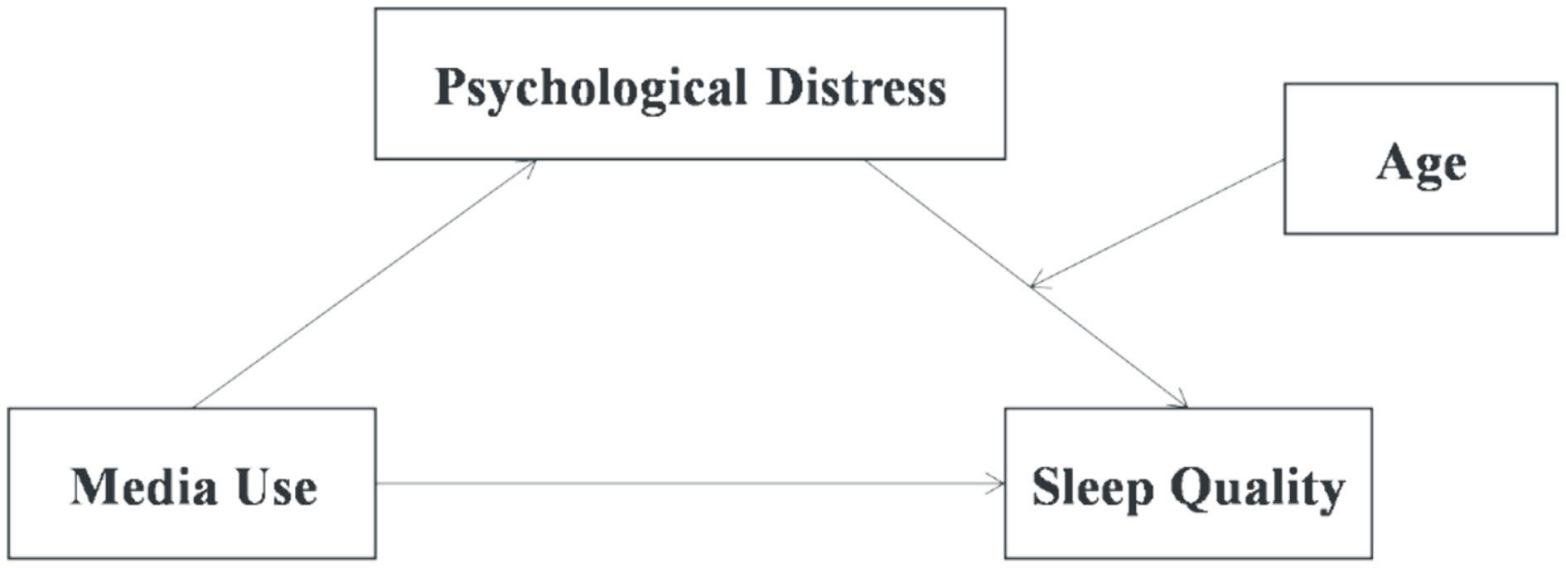
Proposed moderated mediation model. This figure illustrates the proposed model of the current study. Media use relates to sleep via psychological distress. Age moderates the relationship between psychological distress and sleep disturbance.

The moderated mediation model is illustrated in Figure 1.

## 2 Methods and materials

### 2.1 Participants and procedure

An online cross-sectional study design was employed. In June 2020, U.K. participants were recruited on an online platform.^1^ Participants first read the purpose of the study and an informed consent. After providing informed consent, they were instructed to complete a series of questionnaires. They were paid with £1. A total of 1,063 participants were included in the current study, including 385 males (36.2%) and 678 (63.8%) females. Participants’ age was between 18 and 77 years, with an average of 38.85 years.

### 2.2 Measures

#### 2.2.1 Media use

Traditional media use was assessed with the question “During the past 2 weeks, how often were you exposed to news and information about COVID-19 on traditional media such as TV, radio, newspaper?” New media use was assessed with the question “During the past 2 weeks, how often were you exposed to news and information about COVID-19 on social media such Facebook, Twitter, Instagram?.” Participants were asked to respond on a five-point ordinal response scale (1 = never, 2 = occasionally, 3 = sometimes, 4 = often, 5 = very often).

#### 2.2.2 Psychological distress

The 10-item Kessler Psychological Distress Scale (K10; Kessler et al., 2002) was used to measure an individual’s psychological distress level and was widely utilized in research and clinical practice in various countries and regions. It has been validated in the UK, with good reliability and validity (Lantos et al., 2023). The K10 asks about the frequencies experiencing a series of negative emotional states based on a 5-point Likert-type scale ranging from 1 (none of the time) to 5 (all of the time). The total scores would thus range from 10 to 50, which higher values signifying more psychological distress. The internal consistency was 0.93 in the current study.

#### 2.2.3 Sleep disturbance

The Insomnia Severity Index (ISI) was used to detect poor sleep in the past 2 weeks (Morin et al., 2011). Seven items assessed difficulty falling or staying asleep, satisfaction with current sleep pattern, interference with daily functioning, the extent to which others notice their sleep problems, and worry/distress related to sleep problems. Response options ranged from 0 (e.g., not at all worried) to 4 (e.g., very much worried), with possible total scores ranging from 0 to 28. Participants scoring ≥10 were classified as screening positive for insomnia (Morin et al., 2011). The ISI is a scale widely used to assess the severity of insomnia and is commonly employed in clinical and research settings. It has been validated in the UK, with good reliability and validity (Akram et al., 2019). The internal consistency was 0.90 in the current study.

#### 2.2.4 Big-Five personality dimensions

The Big-Five personality dimensions were measured by the Ten-item Personality Inventory (TIPI), a brief self-report questionnaire used to assess Big-Five personality traits: extraversion, agreeableness, conscientiousness, emotional stability, and openness to experience (Gosling et al., 2003). Each personality was measured by two adjectives. Participants rated on a scale ranging from 1 to 7 to indicate to what extant these adjectives can describe themselves. The TIPI has been validated in the UK (Westgarth et al., 2018). In the current study, the internal consistency indices were 0.71, 0.40, 0.58, 0.73, and 0.45 for Extraversion, Agreeableness, Conscientiousness, Emotional Stability, and Openness to Experience, respectively.

#### 2.2.5 Demographics and controls

We collected demographic information, including gender (1 = Male, 2 = Female), age, educational level (1 = Primary school or less, 2 = Lower secondary school, 3 = Upper secondary school; 4 = Junior college, 5 = Bachelor, 6 = Master, 7 = Doctorate), monthly household income (1 = £1,000 or less, 2 = £1,000 – £2,000, 3 = £2,000 – £3,000, 4 = £3,000 – £4,000, 5 = £4,000 – £5,000, 6 = £5,000 – £6,000, 7 = £6,000 – £7,000, 8 = £7,000 – £8,000, 9 = £8,000 – £9,000, 10 = £9,000 – £10,000, 11 = £10,000 or more), and ethnicity (1 = White, 2 = Not White). We also asked how healthy participants thought they were (1 = Very poor, 2 = Poor, 3 = Ok, 4 = Good, 5 = Excellent). Details are shown in Table 1.

**Table 1:** Sample characteristics.

Variable		*n* (%)
Age	18–25	161 (15.1)
	26–40	491 (46.2)
	41–55	257 (24.2)
	>55	154 (14.5)
Gender	Male	385 (36.2)
	Female	678 (63.9)
Education	Primary school or less	1 (0.1)
	Lower secondary school	35 (3.3)
	Upper secondary school	197 (18.5)
	Junior college	205 (19.3)
	Bachelor	455 (42.8)
	Master	154 (14.5)
	Doctorate	16 (1.5)
Ethnicity	White	967 (91)
	Other	96 (9)
Income now	£1,000 or less	102 (9.6)
	£1,000 – £2,000	213 (20)
	£2,000 – £3,000	268 (25.2)
	£3,000 – £4,000	187 (17.6)
	£4,000 – £5,000	89 (8.4)
	£5,000 – £6,000	49 (4.6)
	£6,000 – £7,000	20 (1.9)
	£7,000 – £8,000	15 (1.4)
	£8,000 – £9,000	13 (1.2)
	£9,000 – £10,000	13 (1.2)
	£10,000 or more	94 (8.8)
Healthy	Very poor	8 (0.8)
	Poor	77 (7.2)
	Ok	353 (33.2)
	Good	499 (46.9)
	Excellent	126 (11.9)

### 2.3 Statistical analyses

All data were processed with SPSS statistical software V.23. The significant level was set at 0.05. First, Pearson’s analysis was conducted to examine the correlations of all the investigated variables. Second, model 4 of SPSS macro PROCESS 3.1 (Hayes, 2013) was used to test the mediating roles of psychological distress in the relationship between exposure to information about COVID-19 and poor sleep by generating bias-corrected bootstrap confidence internal (using 5,000 bootstrapping samples). Third, the moderated mediation model was tested with model 14 of SPSS macro PROCESS. When testing mediation and moderated mediation, we also did sensitivity analyses by considering gender, educational level, ethnicity, household income, health status, and personality traits as control variables.

## 3 Results

### 3.1 Testing for common method bias

In this study, data was collected using self-report questionnaires, which may be susceptible to common method bias. Therefore, before conducting data analysis, Harman’s single-factor test was employed to assess the presence of common method bias. The results indicated that the eigenvalues of 10 non-rotated factors were greater than 1, and the variance explained by the first factor was 27.90%, which was below the critical threshold of 40%. Thus, common method bias was not significant in this study.

### 3.2 Descriptive statistics and correlation analysis of variables

Descriptive statistics of all measured variables and correlations among these variables are displayed in Tables 2, 3, respectively. There was a significant positive correlation between new media use and psychological distress as well as sleep disturbance, thus supporting Hypothesis 1. Psychological distress and sleep disturbance were also significantly positively correlated. Age was significantly negatively correlated with new media use, psychological distress, and sleep disturbance, but positively correlated with traditional media use. There was no significant correlation between traditional media use and either psychological distress or sleep disturbance.

**Table 2:** Descriptive statistics of all measured variables.

Variable	Mean	SD	Skewness	Kurtosis
Age	38.85	13.36	0.62	−0.47
Gender	1.64	0.48	−0.57	−1.67
Educational level	4.51	1.10	−0.35	−0.44
Household income	4.10	2.77	1.40	1.12
Ethnicity	1.09	0.29	2.86	6.21
Health status	3.62	0.81	−0.34	0.06
Extraversion	3.48	1.50	0.29	−0.76
Agreeableness	4.91	1.16	−0.32	−0.11
Conscientiousness	5.28	1.20	−0.71	0.24
Emotional stability	4.39	1.44	−0.12	−0.76
Openness	4.61	1.17	−0.22	−0.27
Traditional media use	4.29	1.00	−1.41	1.20
New media use	3.81	1.38	−0.85	−0.65
Psychological distress	18.61	7.68	1.18	1.05
Sleep problem	8.56	5.80	0.65	0.08

**Table 3:** Correlations among all measured variables.

Variable	1	2	3	4	5	6	7	8	9	10	11	12	13	14
1. Age	1													
2. Gender	−0.06	1												
3. Educational level	−0.14**	0.05	1											
4. Household income	−0.12**	−0.01	0.07*	1										
5. Ethnicity	−0.20**	0.02	0.09**	0.00	1									
6. Health status	−0.05	0.03	0.14**	0.18**	−0.02	1								
7. Extraversion	−0.04	0.08*	0.00	0.07*	−0.01	0.18**	1							
8. Agreeableness	0.18**	0.09**	−0.02	0.05	0.01	0.12**	0.026	1						
9. Conscientiousness	0.18**	0.03	0.01	0.04	−0.04	0.26**	0.13**	0.27**	1					
10. Emotional stability	0.16**	−0.16**	0.00	0.04	0.04	0.33**	0.21**	0.29**	0.36**	1				
11. Openness	−0.07*	0.03	0.07*	0.01	0.03	0.13**	0.29**	0.17**	0.16**	0.16**	1			
12. Traditional media use	0.13**	0.02	0.03	0.05	−0.08*	0.05	−0.01	0.09**	0.14*	0.02	−0.04	1		
13. New media use	−0.30**	0.10**	0.08**	0.07*	−0.001	−0.02	0.06*	−0.01	−0.07*	−0.09**	0.05	0.32**	1	
14. Psychological distress	−0.24**	0.03	−0.02	−0.05	0.03	−0.36**	−0.13**	−0.19**	−0.32**	−0.56**	−0.05	−0.01	0.17**	1
15. Sleep problem	−0.13**	0.01	0.00	−0.03	0.03	−0.31**	−0.11**	−0.06*	−0.21**	−0.39**	−0.03	0.03	0.13**	0.64**

*p < 0.05, **p < 0.01, ***p < 0.001.

### 3.3 Testing for the mediation effect of psychological distress

A mediation model was constructed to examine the mediating effect of psychological distress between new media use and sleep disturbance using Model 4 in the PROCESS macro. First, Model 1 in Table 4 showed that new media use (i.e., the predictor) significantly predicted sleep disturbance (i.e., the outcome) (parameter *c* in the mediation analysis), *β* = 0.13, *p* < 0.001. Second, as shown in Model 2 in Table 3, new media use (i.e., the predictor) significantly predicted psychological distress (i.e., the mediator) (parameter *a* in the mediation analysis), *β* = 0.17, *p* < 0.001. Finally, as shown in Model 3 in Table 4, when new media use (i.e., the predictor) and psychological distress (i.e., the mediator) was simultaneously entered, new media use was no longer a significant predictor of sleep disturbance (parameter *c’* in the mediation analysis), *β* = 0.03, *p* = 0.241, whereas psychological distress (i.e., the mediator) was still significant (parameter *b* in the mediation analysis), *β* = 0.63, *p* < 0.001. The bootstrap estimation procedure with 5,000 bootstrapping samples showed that the total effect was 0.56. The indirect effect was 0.44 (78.57% of the total effect), SE = 0.07, 95%CI [0.29, 0.58] and the direct effect was 0.12, SE = 0.10, 95%CI [−0.08, 0.32]. The confidence interval for the direct effect included zero, suggesting that psychological distress fully mediated the relationship between new media use and sleep disturbance. Sensitivity analyses including all covariates found very similar results, as shown in Models 4–6 in Table 4, suggesting the results were robust. Therefore, Hypothesis 2 was supported.

**Table 4:** Regression results of the mediation.

	Model 1		Model 2		Model 3		Model 4		Model 5		Model 6	
	Sleep problem		Psychological distress		Sleep problem		Sleep problem		Psychological distress		Sleep problem	
	*β*	*t*	*β*	*t*	*β*	*t*	*β*	*t*	*β*	*t*	*β*	*t*
Predictors												
New media use	0.13***	4.37	0.17***	5.49	0.03	1.17	0.08**	2.79	0.09**	3.12	0.03	1.38
Psychological distress					0.63***	26.23					0.58***	19.32
Covariates												
Age							−0.07*	−2.19	−0.14***	−5.19	0.01	0.53
Gender							−0.05	−1.79	−0.05	−1.96	−0.02	−0.92
Education							0.02	0.55	−0.01	−0.51	0.02	0.94
Household income							0.00	0.02	−0.02	−0.65	0.01	0.41
Ethnicity							0.02	0.86	0.01	0.48	0.02	0.71
Health status							−0.21***	−6.84	−0.19***	−7.21	−0.10***	−3.58
Extraversion							−0.02	−0.59	−0.01	−0.52	−0.01	−0.37
Agreeableness							0.09**	2.75	0.01	0.34	0.08**	3.00
Conscientiousness							−0.05	−1.61	−0.08**	−3.10	−0.00	−0.03
Emotion stability							−0.32***	−9.64	−0.45***	−15.78	−0.05	−1.63
Openness							0.03	1.15	0.05	1.81	0.01	0.25
*R* ^2^	0.02		0.03		0.40		0.21		0.40		0.42	
*F*	19.08***		30.09***		359.61***		23.77***		57.28***		58.43***	

*p < 0.05, **p < 0.01, ***p < 0.01. *β* is the standardized coefficient.

### 3.4 Testing for the moderating effect of age

**Figure 2:**
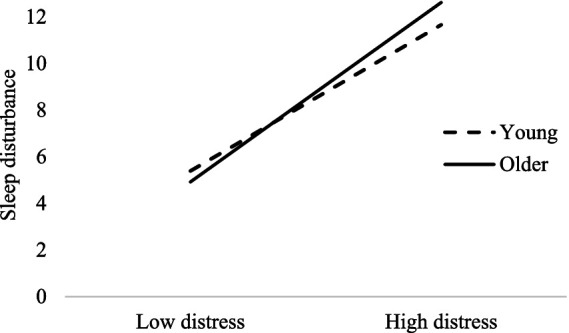
The moderating effect of age on the relationship between psychological distress and sleep problem after controlling for covariates.

To test whether age moderated the relationship between psychological distress and sleep disturbance as well as the mediating effect of psychological distress on the relationship between new media use and sleep disturbance after controlling for covariates, we used Model 14 in the PROCESS macro developed by Hayes (2013) to examine the moderated mediation model. As shown in Model 1 in Table 5, the interactional effect of age and psychological distress on sleep disturbance was significant, *β* = 0.23, *p* = 0.004. Simple slope analysis showed that psychological distress predicted sleep disturbance for older people (1 SD above the mean), *β* = 0.55, *t* = 18.92, *p* < 0.001. For younger people (1 SD below the mean), however, the predictive effect of psychological distress on sleep disturbance was reduced, *β* = 0.44, *t* = 18.58, *p* < 0.001. The pattern is depicted in Figure 2.

**Table 5:** Regression results of the moderation.

	Model 1		Model 2	
	Sleep problem		Sleep problem	
	*β*	*t*	*β*	*t*
Predictors				
New media use	0.03	1.25	0.03	1.20
Psychological distress	0.44***	6.21	0.42***	5.83
Age	−0.14*	−2.18	−0.13*	−2.01
Age × psychological distress	0.23**	2.91	0.20*	2.46
Covariates				
Gender			−0.02	−0.90
Education			0.02	0.76
Household income			0.01	0.55
Ethnicity			0.02	0.63
Health status			−0.10***	−3.46
Extraversion			−0.01	−0.31
Agreeableness			0.08**	2.94
Conscientiousness			−0.01	−0.25
Emotion stability			−0.04	−1.37
Openness			0.01	0.32
*R* ^2^	0.41		0.42	
*F*	183.62***		54.95***	

*p < 0.05, **p < 0.01, ***p < 0.01. *β* is the standardized coefficient.

The bootstrap estimation procedure with 5,000 bootstrapping samples revealed a significant moderated mediation, Effect = 0.004, SE = 0.001, 95%CI [0.001, 0.007]. Specifically, the mediating effect of psychological distress on the relationship between new media use and sleep problem was stronger for older people (1 SD above the mean), Effect = 0.50, SE = 0.09, 95%CI [0.33, 0.67] than for younger people (1 SD below the mean), Effect = 0.40, SE = 0.07, 95%CI [0.27, 0.54]. Sensitivity analyses including all covariates found very similar results, as shown in Models 2 in Table 5, suggesting the results were robust. Therefore, Hypothesis 3 was supported.

## 4 Discussion

In this study, we investigated the influence of media consumption on sleep quality amidst the backdrop of the COVID-19 pandemic by developing a moderated mediation model. Our findings unveiled that the utilization of new media platforms can indirectly impact sleep quality through the pathway of psychological stress. Furthermore, we observed that age plays a moderating role in the relationship between psychological distress and sleep quality. Although the global situation regarding the COVID-19 pandemic has changed since we collected data for this research, our results remain significant. The influence of media, especially new media, is pervasive and enduring. The use of new media tends to result in psychological distress and poor sleep quality not only during the early stages of the COVID-19 pandemic but also during other global or regional crises and even in our everyday lives.

### 4.1 Mediating role of psychological distress

This study highlights that during the COVID-19 era, the utilization of new media is linked to diminished sleep quality, contrasting with traditional media use. This finding is in line with prior research outcomes (Goodwin et al., 2018; Chao et al., 2020). The distinctive attributes of new and traditional media exert different impacts on users. Traditional media channels, such as newspapers, magazines, television, and radio, function as unidirectional mass communication tools, offering information in a readily comprehensible manner that aids in information acquisition, disease awareness, and risk perception, especially for individuals with lower educational backgrounds (Ho, 2012). In contrast, new media, notably social media, empower users to access, generate, and exchange content, fostering the dissemination, evaluation, and discourse of information (Yu and Chen, 2021). Social media platforms like Facebook, YouTube, and Twitter enable interactive engagement, facilitating active commenting and sharing of crisis-related information, thereby enhancing the likelihood of other users accessing this information. However, this interactive nature may result in a lack of thorough scrutiny of crisis information by the public, potentially fuelling the spread of misinformation or panic, heightening fear and anxiety (Abd-Alrazaq et al., 2020).

Amid the COVID-19 pandemic, internet and social media usage surged to unprecedented levels (Marzouki et al., 2021). Users consumed a greater volume of COVID-19 information through social media, seeking the latest updates (Kaya, 2020). Excessive focus on event-related information during traumatic occurrences can exacerbate psychological distress (Li and Khan, 2022). Studies also indicate that individuals engaged in social media platforms experience heightened post-traumatic stress compared to those relying solely on traditional media, as the content shared on new media platforms can directly and personally impact individuals (Goodwin et al., 2013). The extensive coverage of COVID-19 by new media may prompt individuals to perceive elevated threat levels, subsequently escalating stress levels (Hwang et al., 2021). Moreover, given the relatively high mortality rate and potential long-term implications of COVID-19, the general populace easily succumbs to virus-related fear (Wang C. et al., 2020; Wang D. et al., 2020). The deluge of information during the pandemic can induce fear and stress, leading to psychological distress (Diotaiuti et al., 2023). In this study, psychological distress emerges as a predictor of diminished sleep quality, with its substantial mediating effect underscoring its significance as a risk factor for sleep disturbance, aligning with prior research (Duran and Erkin, 2021; Olagunju et al., 2021). When an individual undergoes psychological distress, their brain becomes stimulated and aroused, impeding rest and culminating in insomnia and other sleep-related issues (Kuriyama et al., 2010). This underscores the importance of monitoring the accuracy of information disseminated on social media and regulating the duration of information exposure to mitigate individuals’ psychological distress.

These findings provide critical insights for policymakers, particularly in anticipation of future health crises. We recommend the following policy and practical applications: Firstly, public health authorities should implement targeted health communication strategies to mitigate the dissemination of inaccurate or distressing information via new media, which has been linked to psychological distress and sleep issues. For instance, establishing rapid response teams to monitor and correct misinformation related to health crises could be beneficial. Secondly, special support services for the elderly should be provided to help them better filter and understand new media content. This can be achieved through community workshops, technical support hotlines, or creating age-friendly online resources. Thirdly, in clinical practice, healthcare providers and mental health professionals should enhance counseling and guidance on the relationship between new media usage and sleep health. For patients presenting with sleep difficulties, practitioners should assess their media usage and offer behavioral management strategies as needed. Lastly, over the long term, governments should consider developing media literacy education programs as part of public health strategies to enhance the public’s psychological resilience and ability to filter information during health crises. Recognizing the impact of new media on public psychological well-being and sleep quality, and addressing these effects through targeted policies and interventions, will be crucial in preparing for future similar crises. We hope that discussions based on these findings will inspire the development of more effective policies and promote public health and well-being.

It should also be noted that in the current study, the UK has a relatively comprehensive social support system, including public healthcare and welfare systems. However, during the pandemic, the National Health Service (NHS) in the UK faced significant challenges, such as canceled surgeries, shortages of medical resources and staff, resulting in longer patient waiting times and decreased service quality, highlighting the vulnerability and challenges of the NHS during crises (Khan, 2023). These circumstances may impact individuals’ mental health and increase their use of new media, as people may seek support or information on social media platforms. Accordingly, public health policies and interventions should take these social context factors into account to effectively alleviate the negative impacts of new media.

### 4.2 Moderating role of age

Our research findings suggest that age acts as a moderator in the relationship between new media usage and sleep quality through its influence on individual psychological distress, with the effect intensifying with advancing age. Particularly, older adults are more susceptible to this influence compared to younger individuals, highlighting a significant finding of this study. Previous research indicates that social media is more likely to impact the sleep quality of adolescents (Mo et al., 2021; Zhang et al., 2021). The differences between these two distinct research findings may stem from variations in social media usage habits or psychological characteristics across different age groups. Bailey et al. (2022) proposes that due to frequent social media exposure, young individuals encounter a vast array of pandemic-related information, some of which are crucial, while some are distressing, resulting in heightened COVID-19-related psychological distress. However, an increasing body of research indicates that older adults report elevated levels of psychological distress (Qiu et al., 2020; Zhang et al., 2021). The perceived ageism and social isolation prevalent among this demographic significantly contribute to the association between age and psychological distress (Berg-Weger and Morley, 2020). A recent study by Petru et al. (2021) reveals that older adults tend to hold more negative attitudes toward COVID-19. Additionally, some studies indicate that as age increases, the prevalence of sleep issues becomes more common (Sonnega and Sonnega, 2018) due to various influencing factors such as neuronal loss and atrophy, reduced cerebral blood flow, and neurotransmitter deficiencies (Polo-Kantola, 2011). As individuals age, older adults may also experience significant life changes such as bereavement, chronic illness, retirement, and the loss of family members. These life events may lead to psychological distress and emotional upheaval, subsequently exerting negative effects on the sleep of older adults (Thomas et al., 2017). Older adults may be more susceptible to sleep disturbances due to being in a higher stage of life transition. Psychological stress and negative emotions may hinder effective coping and adjustment among older adults, impacting their sleep quality (Zhang et al., 2024). Therefore, understanding these characteristics is of significant importance for the psychological well-being and sleep management of the elderly population.

### 4.3 Limitations

First, the cross-sectional design of this study cannot establish causality. Future research should adopt a longitudinal approach to provide a more dynamic and comprehensive understanding of how the psychological effects of a global crisis, such as the COVID-19 pandemic, evolve over time. Second, the moderating effect of age in this study was relatively small, which needs further empirical research for confirmation. Third, there are several factors hampering the generalizability of our results: (1) this study used an online survey, which, while currently the best solution for collecting data during social distancing times, limits the representativeness of the sample; (2) this study only measured individuals’ media use levels through one question, and it is unclear how participants accessed, where they accessed, and what COVID-19 information they obtained; (3) we relied on self-report surveys, which may be limited by subjectivity and memory biases; and (4) although we controlled several demographic factors, there may be other confounding variables influencing the relationship we found.

## 5 Conclusion

In conclusion, the moderated mediation model developed in this study elucidates the pathway through which new media usage impacts sleep quality during the COVID-19 pandemic. The inclusion of moderating effects highlights variations in the intensity of the mediation effects across individuals of different age groups. This model offers a more profound understanding of how new media use affects sleep quality: new media use indirectly influences sleep quality through psychological distress, with the moderation by age influencing the latter part of this process.

## Data availability statement

The datasets presented in this study can be found in online repositories. The names of the repository/repositories and accession number(s) can be found at: https://osf.io/m8rtb/?view_only=48b08afc19e043cb9eb5462626c7d7a7.

## Ethics statement

The studies involving humans were approved by Department of Psychology, Jianghan University. The studies were conducted in accordance with the local legislation and institutional requirements. The participants provided their written informed consent to participate in this study.

## Author contributions

TX: Investigation, Writing – original draft. DZ: Conceptualization, Supervision, Writing – original draft. ZY: Investigation, Supervision, Writing – review & editing. JD: Conceptualization, Formal analysis, Supervision, Validation, Writing – original draft, Writing – review & editing. HS: Supervision, Writing – review & editing.

## Footnotes

^1^https://www.prolific.co/
